# Quantitative Measurements of Backside Wear in Acetabular Hip Joint Replacement: Conventional Polyethylene Versus Cross-Linked Polyethylene

**DOI:** 10.3390/ma13081854

**Published:** 2020-04-15

**Authors:** Steffen Braun, Sebastian Jaeger, Robert Sonntag, Stefan Schroeder, J. Philippe Kretzer

**Affiliations:** Laboratory of Biomechanics and Implant Research, Heidelberg University Hospital, Schlierbacher Landstraße 200a, 69118 Heidelberg, Germany

**Keywords:** backside wear, cross-linked, total hip replacement, hip cup system

## Abstract

As shown in previous studies, the modification of conventional polyethylene (CPE) to cross-linked polyethylene (XLPE) and the contribution of antioxidants result in a reduction in total wear. The aim of this study was to evaluate XLPE inserts with vitamin E and CPE regarding their resistance to the backside wear mechanism. A cementless hip cup system (Plasmafit^®^ Plus 7, Aesculap) was dynamically loaded using CPE and XLPE inserts. The backside wear was isolated, generated and collected using the two-chamber principle. The chambers were filled with ultrapure water. After 2 × 10^6^ cycles, the fluids were examined for wear particles according to a particle analysis. Using XLPE inserts, the backside wear was significantly reduced by 35%. While XLPE backside wear particles are significantly larger than CPE particles, they do not differ in their morphology. This study confirms the greater resistance to backside wear of XLPE compared to CPE. It can be assumed that the improved fatigue resistance of the vitamin E-stabilized XLPE inserts demonstrates XLPE’s effectiveness against micro-motion and the resulting changing tensions in interface areas like surface breakdown, pitting and the release of very small particles.

## 1. Introduction

The modification of conventional polyethylene (CPE) to cross-linked polyethylene (XLPE) and the introduction of antioxidants results in a reduction in joint articular wear [1,2,3].

While CPE has been the gold standard in hip joint replacement for many years, it is being increasingly replaced by XLPE at a rate of over 95% in hip arthroplasty [4,5]. There is a continuing trend towards bearings with ceramic heads and XLPE inserts [4,5]. Polymer chains that are cross-linked as a result of defined gamma or electron radiation in oxygen-free settings and subsequent thermal treatment show a higher articular wear resistance compared to CPE [6]. In addition, antioxidants such as vitamin E are used to bind free radicals in order to improve the mechanical stability, fatigue strength and oxidation resistance of XLPE [7], which results in a further reduction in articular wear [1,2,3]. Furthermore, XLPE with vitamin E shows an effective prevention of oxidation even with long aging and leads to more consistent wear behavior compared to CPE and XLPE [8].

Established and internationally standardized examination methods are frequently used to evaluate the articular wear resistance of joint replacements. In addition, a new investigation method enables the isolated quantitative measurement of polyethylene (PE) backside wear in modular acetabular cups [9]. While the resistance to articular wear processes of XLPE has already been demonstrated in many experimental [1,10,11,12,13] and clinical studies [3,14,15], no quantitative evidence exists about the supposed wear advantage of XLPE in terms of backside wear. 

Therefore, the aim of this study was to compare the backside wear behavior of CPE and XLPE inserts. The questions were defined as follows: Is less PE backside wear generated by using XLPE than using CPE? Is there a difference in the size and morphology of the backside wear particles of XLPE compared to those of CPE?

## 2. Materials and Methods

### 2.1. Experimental Groups

Two test groups were compared to each other (CPE versus XLPE). Each group included three cups and three inserts, which were tested independently of each other under the same test conditions.

### 2.2. Analyzed Components

The cementless cup system Plasmafit^®^ Plus 7 (Aesculap, Tuttlingen, Germany, Ref: NV352T) with a cup size of 52 mm was used. The locking mechanism for fixing the PE inserts was based on a conical width and rough striking surface (R_a_ = 3.7 µm, R_z_ = 24.7 µm). PE inserts with an inner diameter of 32 mm were used for all investigations. For the first group (CPE), conventional PE (Ref: NV203) in accordance with ISO 5834-2 was used. Cross-linked PE Vitelene^®^ (Aesculap, Tuttlingen, Germany, Ref: NV203E) with vitamin E as the antioxidant was used for the second group (XLPE). 

### 2.3. Test Setup

The method used for the quantitative measurement of PE backside wear has been validated and described in detail [9].

**Figure 1:**
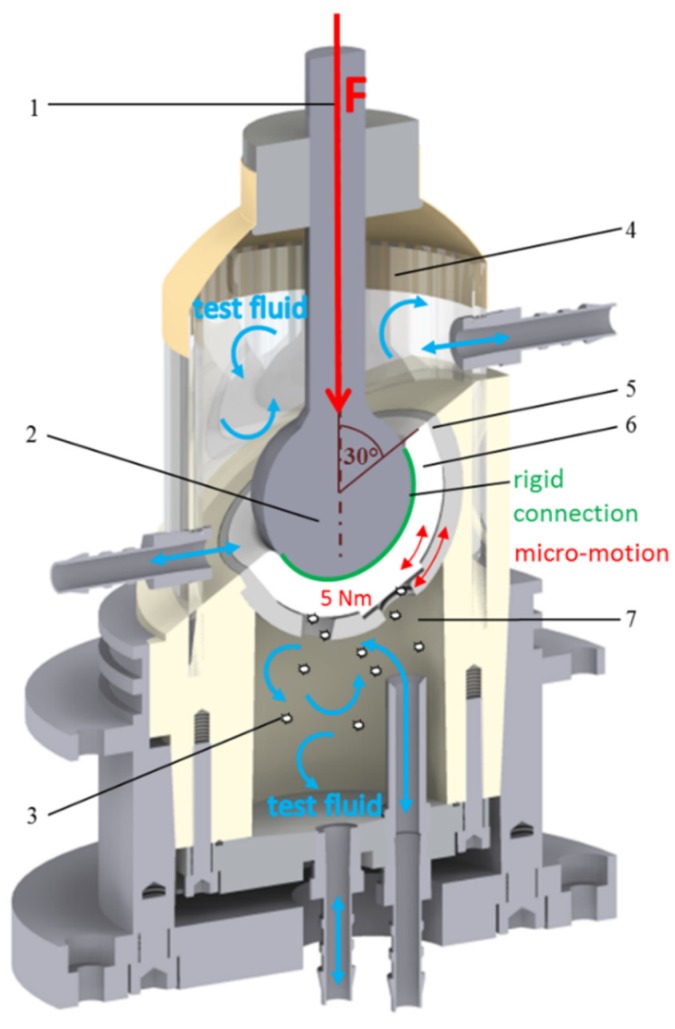
Test setup according to the two-chamber principle: (**1**) load axis, (**2**) artificial femoral head, (**3**) backside wear particle, (**4**) articulation area, (**5**) cup system, (**6**) PE insert, (**7**) backside area [9].

The cup system (Figure 1(5)) was fixed in polyurethane and dynamically loaded with a frequency of 3 Hz and a force of up to 2.5 kN. Simultaneously, a moment of about 5 Nm (Figure 1) was induced in the cup system. The inclination of the cup was 30° to the load axis (Figure 1(1)). The test duration was 2 × 10^6^ cycles. 

According to the two-chamber principle, the backside area (Figure 1(7)) was separated from the articulation area (Figure 1(4)) and each area represented a chamber system. Both chambers were filled with ultrapure water as the test fluid. However, the interface between the PE insert and the cup could not be considered a reliable seal between the articulation area and the backside cup area. To ensure an isolated generation of backside wear particles, the articulation of the sliding partners (head and PE insert) and thus the articular wear must be prevented. Due to the rigid and resistant cohesive connection (Loctite^®^ 406/SF770, Henkel AG & Co. KGaA, Duesseldorf, Germany) (Figure 1) between the articulation surfaces (femoral head and insert (Figure 1(2,6)), an isolated generation of insert backside wear (Figure 1(3)) was provided. Thereby, backside-generated PE particles were collected in the test fluid. The integrity of the rigid connection was checked and confirmed before and after each test. 

### 2.4. Wear Analysis

After the test was completed, the test fluid was removed from the chambers and analyzed for wear particles. For this analysis, the test fluid was vacuum filtered (pore size: 0.02 µm) and the filters were examined by a scanning electron microscope (SEM Leo 1530, Carl Zeiss AG, Oberkochen, Germany) at a 20,000 times magnification (3 filters/3 SEM images per chamber). The SEM images created were then analyzed using image processing software (ImageJ, version 1.48, public domain) and a particle analysis was carried out in accordance with ASTM F1877-16 [16]. The wear particles were characterized in terms of their size (ECD) and morphology (aspect ratio (AR) and roundness (R)). The particle shapes were characterized as round, oval and fibril-like according to their AR [17]. In addition, the number of analyzed particles was extrapolated to obtain a total number of particles (ETN) as a measure of PE wear [9]. 

### 2.5. Statistics

Descriptive statistics with the mean and standard deviations of nine individual values were given for all results. An independent t-test was used to compare all the mean parameters (ETN, ECD, AR and R) of the particle analysis of the two groups. All the requirements for the implementation of statistical procedures were confirmed. The software SPSS (Version 22, IBM, Amonk, NY, USA) was used for the statistical evaluation. The level of significance was set at 5% (*p* < 0.05). 

## 3. Results

**Figure 2:**
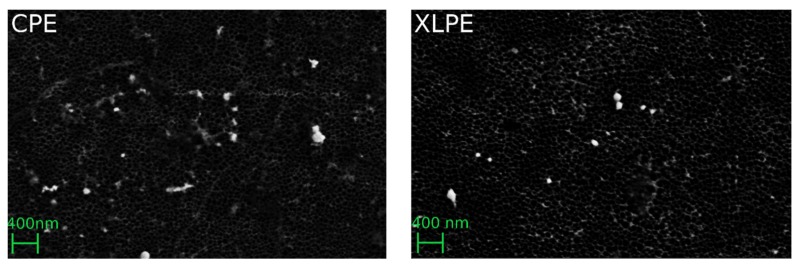
Filter images of the analyzed backside PE wear particles.

**Figure 3:**
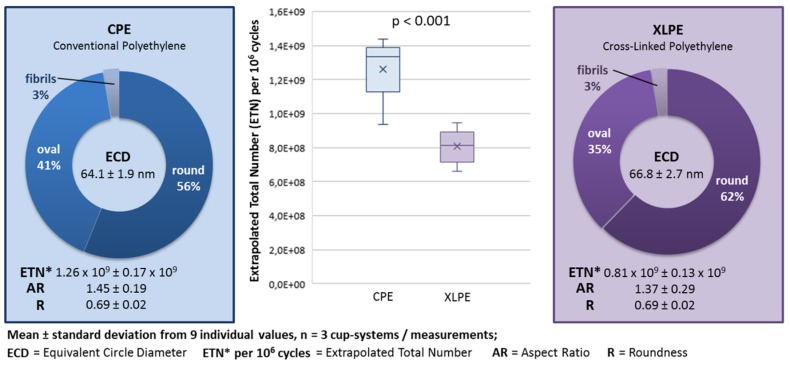
Results of the particle analysis and comparison between the conventional polyethylene (CPE) and cross-linked polyethylene (XLPE) backside wear.

Figure 2 shows an example of isolated backside PE wear particles. Figure 3 shows the mean and standard deviations of the PE backside wear in a direct comparison between the use of CPE and XLPE in the investigated cup system. The results of the CPE backside wear with the parameters ETN, ECD, AR and R were partially published in a previous study [9]. In addition, the amounts of different particle shapes and the median (max and min) are shown in Figure 3.

Using the XLPE, about 35% less PE backside wear was generated compared to when the CPE was used. This difference was statistically significant (ETN: *p* < 0.001). 

**Figure 4:**
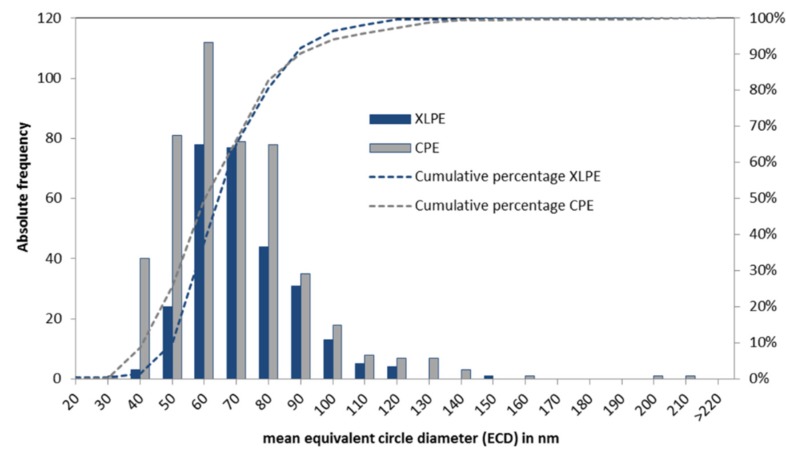
The size distribution of the analyzed particles from the CPE and XLPE inserts.

The morphology parameters AR and R did not differ significantly between the CPE and XLPE (AR: *p* = 0.465 and R: *p* = 0.126). The particles were predominantly characterized by a round morphology. The XLPE tended to have a larger amount of round particles than the CPE. In addition, the XLPE particles were slightly but significantly larger (*p* = 0.028). The PE backside wear particles were generally nanoparticles in a size range of between 40 and 100 nm (Figure 4). 

**Figure 5:**
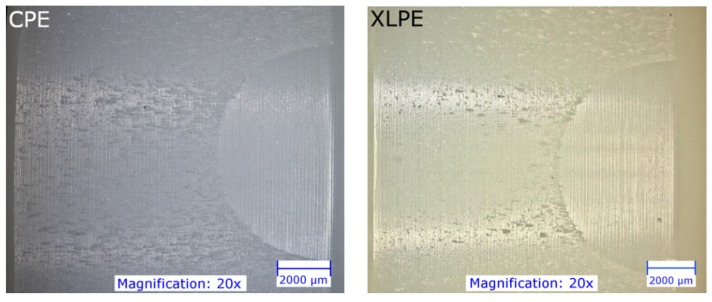
Photographs (Digital Microscope VHX-5000, Keyence, Japan) of the backside of the tested PE inserts.

Figure 5 shows an example of the backside of the CPE and XLPE inserts. It seems that micro-scratches are much more pronounced on the CPE inserts than on the XLPE inserts, whereas the XLPE inserts show a higher proportion of pitting. However, the damage to the XLPE inserts is generally less pronounced. 

## 4. Discussion

The use of XLPE generated significantly less PE backside wear than the use of CPE for the investigated cup system. With the CPE and XLPE inserts, predominantly round particles (56–62%) were generated on the backside and the XLPE particles were significant larger.

The particle analysis of the reported articular wear showed comparable results regarding the morphology [10,12,18]. Illgen et al. described predominantly round particles from CPE (amount: 78%) and from XLPE (amount: 87%) [10]. The amount of elongated fibrils was significantly higher for CPE (14%) than for XLPE (6%). In addition, the CPE particles in published articular wear studies (approx. 196–710 nm) are significantly larger than the XLPE particles (approx. 110–260 nm) [10,12,13]. There was a significant difference in the size of backside-generated particles in this study. The difference in size between the CPE and XLPE particles was only around 3 nm. Considering the size distribution (about 40–100 nm) of the analyzed PE particles, the difference seems negligibly small. However, this 3 nm was a difference of 4%. Fewer wear particles that are of a larger size could result in a higher volume of wear. However, the significantly higher amount of wear particles generated by the CPE inserts (by 35%) seems to have been more relevant than the 4% larger size of the XLPE particles. In addition, we only have two-dimensional images of the particles, which makes it impossible to determine the volume of wear. 

The reason for the comparable particle shape was probably the rough locking surface of the cup. The rough peaks of the titanium locking surface engaged the soft PE insert. Due to the micro-motion at this interface, the rough peaks plowed grooves into the soft surface layer of the PE. Therefore, the resulting wear mechanism was dominated by scratching and micro-machining and thus produced comparable wear particles in terms of size and shape. This could be confirmed by the similar type of damage on the backside of the CPE and XLPE inserts. In addition, the permanent alternating stress could cause material fatigue in the PE interface areas and could lead to the release of very small particles.

The higher articular wear resistance of XLPE has not only already been demonstrated in some experimental studies [1,10,11,12,13], but also in clinical trials [3,14,15,19,20], which examined the primary wear process. In vitro wear simulating showed a great decrease in wear rate, significantly higher fatigue resistance and improved mechanical properties for XLPE compared to CPE inserts [1,8,11]. In this study, the XLPE also showed greater resistance to backside wear. An explanation for this might be that the improved fatigue resistance of the vitamin E-stabilized inserts was effective against the occurring micro-motions and resulting variations in stress in the PE interface areas (surface disruption, pitting and release of very small particles). 

In addition to lower total wear, previous studies have documented a significantly lower occurrence of osteolysis and lower revision rate in patients with XLPE components [3,15,21]. Hanna et al. described a survival rate of 86% for CPE and 100% for XLPE after 13 years with revisions due to excessive wear or osteolysis [15]. While osteolysis was identified in patients with CPE in up to 25% of cases, osteolysis could only be detected in about 2% of cases in patients with XLPE [3,15,21]. According to Fukui et al., patients with XLPE had no wear rate above the osteolysis threshold of 0.1 mm/year, in contrast to 35% of cases in patients with CPE [3]. Cellular responses to the wear particles of XLPE are controversial. While no cell responses [22] or significantly reduced functional biological activity [23] were detected in patients with XLPE in some studies, other studies have shown a significantly larger inflammatory response to XLPE particles [10,13,24,25] than to CPE particles. Illgen et al. showed a concentration-dependent inflammatory response of macrophages against CPE and XLPE particles [10]. At low concentrations, no significant differences in the biological inflammatory response could be observed between CPE and XLPE. However, a significantly higher response was shown to XLPE particles at higher particle concentrations than to CPE wear particles [10]. This higher inflammatory response with a high particle concentration could certainly play an important role with regards to pelvic lysis behind the cups. A particle migration within the cup system [26] could lead to a local accumulation of wear products in the area of screw holes on the bony acetabulum and thereby steadily increase the concentration of PE particles. Therefore, the migration of articulating and backside-generated PE particles should be avoided as far as possible [26]. 

## 5. Limitations

This study was an experimental investigation. Deviations from the clinical situation were unavoidable. Therefore, the following limitations must be taken into account.

Instead of bovine serum or a similar lubricant used for in vitro wear studies in accordance with ISO 14242, ultrapure water was used as the test fluid. However, the backside wear mechanism was completely different to the articulation wear mechanism. On the backside, the rough peaks of the harder inner surface of the cup plowed grooves into the surface of the softer PE. Therefore, the influence of the lubricant on the backside wear mechanism was assumed to be negligible. In addition, ultrapure water proved to be advantageous due to its simpler handling and lower risk of contamination by proteins or other residues of biological substances. On the basis of the structure of the artificial cup system, a reliable hermetic sealing of the articulation area from the backside of the PE insert could hardly be achieved. Therefore, articulation between the head and PE insert was prevented (due to the rigid connection). Subsequently, the usual articulation between the sliding bearings was not possible and the applied force represented a simplified load condition compared to physiological hip loads. However, together with the introduction of moments, the load situation approximated the clinical situation in the cranial–caudal axis [27]. 

## 6. Conclusions

XLPE showed a significantly higher backside wear resistance compared to CPE. It can be assumed that the improved fatigue resistance of the vitamin E-stabilized XLPE inserts demonstrated XLPE’s effectiveness against the occurring micro-motion and resulting changing tensions in and below the interface areas like surface breakdown, pitting and the release of very small particles. The detected backside wear particles were smaller than the reported particles of articular wear, while the morphology of the backside-generated CPE particles did not differ from those of the XLPE.
